# Knowledge of Glasgow coma scale by air-rescue physicians

**DOI:** 10.1186/1757-7241-17-39

**Published:** 2009-09-01

**Authors:** Catherine Heim, Patrick Schoettker, Nicolas Gilliard, Donat R Spahn

**Affiliations:** 1 Department of Anesthesiology, Centre Hospitalier Universitaire Vaudois (CHUV), Lausanne, Switzerland; 2 Department of Anesthesiology, University Hospital, Zurich, Switzerland

## Abstract

**Objective:**

To assess the theoretical and practical knowledge of the Glasgow Coma Scale (GCS) by trained Air-rescue physicians in Switzerland.

**Methods:**

Prospective anonymous observational study with a specially designed questionnaire. General knowledge of the GCS and its use in a clinical case were assessed.

**Results:**

From 130 questionnaires send out, 103 were returned (response rate of 79.2%) and analyzed. Theoretical knowledge of the GCS was consistent for registrars, fellows, consultants and private practitioners active in physician-staffed helicopters. The clinical case was wrongly scored by 38 participants (36.9%). Wrong evaluation of the motor component occurred in 28 questionnaires (27.2%), and 19 errors were made for the verbal score (18.5%). Errors were made most frequently by registrars (47.5%, p = 0.09), followed by fellows (31.6%, p = 0.67) and private practitioners (18.4%, p = 1.00). Consultants made significantly less errors than the rest of the participating physicians (0%, p < 0.05). No statistically significant differences were shown between anesthetists, general practitioners, internal medicine trainees or others.

**Conclusion:**

Although the theoretical knowledge of the GCS by out-of-hospital physicians is correct, significant errors were made in scoring a clinical case. Less experienced physicians had a higher rate of errors. Further emphasis on teaching the GCS is mandatory.

## Introduction

The Glasgow Coma Scale (GCS) was developed more than thirty years ago as a practical tool to measure the "depth and duration of impaired consciousness" [1]. Simplicity was the principle concern with the goal to provide a method to quantify and communicate reliable information about level of consciousness. Thirty years after its initial publication, it has reached worldwide acceptance for assessment and description of patients with neurological impairment [2-4]. In the out-of-hospital setting, the GCS is an important tool for decision-making and triage and its initial score acts as an important prognostic indicator after traumatic brain injury (TBI) [5-8].

The correct assessment of the GCS shows variability among providers and it's assessment has been shown to be difficult with variable implications on treatment [9-11]. Patients on scene are often unstable and more difficult to assess [12]. The reliability of GCS scoring is thus particularly important in this context as it is used to make airway management and disposition decisions [13]. The out-of-hospital GCS is also of value for the attending neurosurgeon and emergency physician when an emergency department GCS cannot be obtained, due to endotracheal intubation and/or neuromuscular paralysis [14]. Inaccurate reporting may result in unnecessary treatment and diagnostic tests. In addition to the summed value, each component of the three categories of the GCS should also be reported [15].

In Switzerland, as in most Europeans countries, out-of-hospital trauma care is provided by physicians on board helicopters or fast-response cars [16,17]. The qualifications of the on-board physicians vary between registrars to consultants and their specialty may be anesthesia, general medicine, internal medicine or others.

The purpose of this study is to assess the knowledge among Swiss air-rescue physicians of the Glasgow Coma Scale by using a specially designed questionnaire.

## Materials and methods

A questionnaire to assess GCS knowledge (additional file 1) was designed by the two first authors of the study. It consisted of two parts:

1. Questions of general nature about the physicians' training and the GCS

Level of training/number of years of practice

Participant's specialty

Familiarity of the participants with the GCS

Knowledge and description of its structure

Knowledge and description of its individual components

2. Description of a clinical scenario

Assessment of a patient having sustained a traumatic brain injury with questions about his GCS and the number of points per component

The medical director of each of the 18 helicopter-bases of the Swiss air-rescue system received a phone call from the first author explaining the purpose and the method of the study. The questionnaires were then sent with a cover letter to the medical director who mailed them to every physician working for his organization in May 2004. Each participant was asked to answer the questionnaire without help and in less than 10 minutes. After completion, the questionnaire was sent back anonymously to the medical director who then returned them to the first author within one month. A reminder phone call was made by the first author two weeks after having sent the questionnaire. Participants were defined as registrars if they were in training, fellows if they had completed their training and were specializing in a sub-specialty, consultants if they were qualified specialists with teaching positions and in private practice if they had left the hospital setting and worked as independent specialists.

### Statistical analysis

Data were analyzed using the JMP 6 statistical package (SAS Institute, Inc, Cary, NC). We used the Fisher's exact test to determine significance between subgroups by rater experience (registrar, fellow, consultant and private practice) or physician's specialization (anesthesia, internal medicine, general practice, others). Data were assessed as non parametric and thus indicated as median [25^th^-75^th ^percentiles]. Results were considered statistically significant when p < 0.05.

## Results

A total of 130 questionnaires were sent to the medical directors of the 18 helicopter bases. Two of the helicopter bases did not participate. The overall response rate from the 16 participating helicopter bases was 79.2% (103 questionnaires). All of the questionnaires returned were complete without any missing data. None of the answered questionnaires had to be excluded.

The Swiss helicopters are staffed mainly by registrars and fellows, mostly with an anesthetic background (table 1). The median clinical experience of all participants is 9.0 years with the anesthetists being the most junior in their training. Median clinical experience of registrars is 5.5 years [IQR 4-7], while the other categories have a median experience of 10 years or more (table 2).

**Table 1:** Demographic data

	**n**	**%**
Questionnaires sent	130	100

Questionnaires returned	103	79.2

Grade		

Registrar	40	38.8

Fellow	36	35.0

Private practice	19	18.5

Consultant	8	7.7

Specialty		

Anesthesia	63	61.2

General medicine	19	18.5

Internal medicine	17	16.5

Others	4	3.9

**Table 2:** Clinical experience of participants by specialty and by grade

**Mean clinical experience by specialty**	**Years, median**	**IQR [25-75]**
Anesthesia	8.0	5.0 -10.25

General Medicine	10.0	8 -19

Internal Medicine	10.0	7 -12

Others	12.0	4.75 -25.25
		
**Mean clinical experience by grade**	**Years, median**	**IQR [25-75]**

Registrar	5.5	4 -7

Fellow	15.5	11.75 -18.75

Consultant	10.0	8 -12

Private practice	15.0	10 -19.5

All of the participating physicians knew about the GCS and were aware of its three components; six physicians (5.8%) incorrectly named one or two components (one error for the eye component, two errors for the motor and four errors for the verbal component) and four participants (3.9%) attributed the wrong number of points to them. The minimal score was described as 3 by 100% of the participants and the maximum score of 15 by all but one physician (99%).

**Figure 1:**
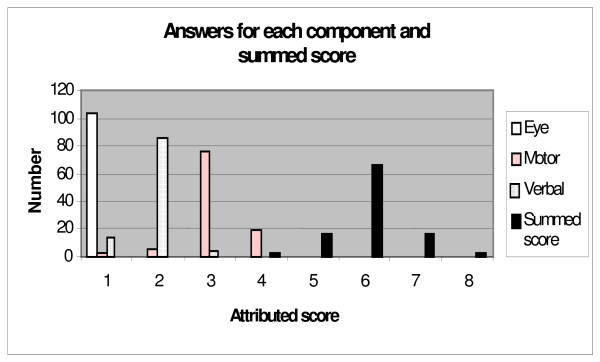
**Range of answers for the clinical case**. Correct value for eye-score: 1. Correct value for motor-score: 3. Correct value for verbal-score: 2. Correct value for summed score: 6.

The clinical case showed incorrect scoring of the overall GCS by 38 physicians (36.9%). While the correct answer was a summed GCS-score of 6, the answers ranged from 4 to 8 (figure 1).

The errors in the assessment of the clinical case by the level of training are shown in table 3. Registrars accounted for 38.8% of the helicopter physicians and were responsible for 50.0% of the total errors. In comparison with fellows, consultants and private practitioners, registrars tended to make more errors (p = 0.095). Among the anesthetists, registrars made significantly more errors in the case analysis (p = 0.039) than their more experienced colleagues of the same specialty. The private practitioners (18.5% of the helicopter physicians) were responsible of 18.4% of the errors (p = 1.00), whereas the fellows (35.0% of the helicopter physicians) made 31.6% of the errors in the clinical scenario (p = 0.671). The consultants made significantly less errors in assessing the clinical case (0%, p < 0.05).

**Table 3:** Distribution of errors in assessment of clinical case by grade and specialty

**Grade**	**Specialty**	**n**	**Errors in numbers**	**Errors in %**
**Registrar (n = 40)**	Anesthesia	29	15	51.7

	Internal medicine	4	2	50

p = 0.095*	General practice	6	1	16.7

	Others	1	1	100

	Total	40	19	47.5
				
**Fellow (n= 36)**	Anesthesia	27	10	37

	Internal medicine	7	1	14.3

p = 0.671*	General practice	1	0	0

	Others	1	1	100

	Total	36	12	33.3
				
**Consultant (n= 8)**	Anesthesia	7	0	0

	Internal medicine	1	0	0

p = 0.025*	General practice	0	0	0

	Others	0	0	0

	Total	8	0	0
				
**Private practice (n= 19)**	Anesthesia	0	0	0

	Internal medicine	5	2	40

p = 1.00*	General practice	12	5	41.7

	Others	2	0	0

	Total	19	7	36.8

*: p-value of each individual grade-category versus the others

The percentage of errors in assessing the clinical case also varied among the different specialties without attaining statistically significant values. Anesthetists assessed incorrectly the case in 39.7%, physicians of internal medicine in 29.4%, those of general medicine in 31.6% and others in 50%.

**Figure 2:**
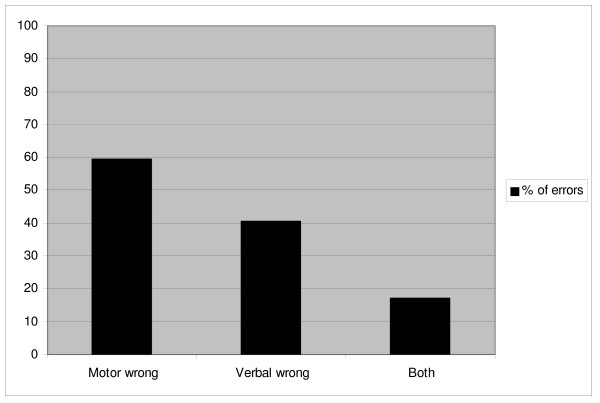
**Distribution of errors in the wrong evaluation of components of the clinical case**.

Differences in assessing the three individual components of the clinical scenario were noted. The motor component score was assessed incorrectly in 28 cases (27.2%), the verbal component in 19 cases (18.5%), while the ocular score was always assessed correctly. Errors were made in assessing both the motor and the verbal component in 8 cases (7.8%) (figure 2). Registrars tended to make more errors in assessing the motor score (p = 0.07) in comparison with fellows (p = 0.818), consultants (p = 0.104) and private practitioners (p = 0.582). Again there was no statistically significant difference in assessing the motor score among subgroups of specialty and level of training.

## Discussion

This study shows that although the GCS is a commonly used tool to assess level of consciousness, more than one-third of air-rescue physicians in Switzerland imprecisely scored it, making errors essentially in the assessment of the motor response.

This is the only study in the medical literature investigating knowledge of the GCS among trained out-of-hospital physicians. So far, most of the studies evaluating the assessment of the GCS were investigating groups of trained versus untrained staff, paramedics versus physicians or nurses. Our study compares physicians working in the emergency air-rescue-system of Switzerland.

All of the participants had proper theoretical knowledge of the GCS. This validates the wide application and theoretical knowledge of the score in the out-of-hospital setting.

We observed an incorrect assessment of the GCS in the clinical scenario by 36.9% of the participating physicians. Errors were associated with level of training, with registrars being responsible for 50% of all errors. This reached statistical significance in the anesthesia group when compared with their more experienced colleagues.

Consultants, who have teaching positions in the Swiss system, made significantly less errors in scoring the clinical scenario. A German study by Lackner et al. analyzed different cohorts of emergency medical staff including physicians, medical students and paramedics on scoring the GCS in video-sequences [18]. They concluded that the level of medical education and professional exposure to trauma patients had a major impact of the accuracy of scoring neurological impairment. Our study included only graduated medical physicians and all of them are regularly exposed to trauma patients.

None of the different specialties investigated was prone to make significantly more errors in the clinical case than another, independently of their level of training.

Previous studies have described variability in the difficulty of scoring the three components of the GCS. [19-21] In this study, the eye component was correctly scored by all participants. This might be consistent with the findings of other authors, who described best accuracy in very high or very low scores [11]. The motor component, with its 6 possibilities, has been shown the most difficult to assess. Among others, the way of eliciting motor response is prone to debate and authors report limb rather than central stimulation. We used truncal stimulation as this was standard at the time of the study. This has been changed since then. Growing evidence suggests that the motor component alone could prove useful for predicting outcome and accurately triaging patients in the trauma setting [8,22-24]. In our study errors in assessing the latter component were responsible for more than half of the errors: again, the lower the experience level of the physician, the more prone to errors. The number of errors in scoring the total GCS of the clinical case was lower than the errors made in scoring the components individually. This indicates the imprecise nature of the summed score.

A limitation of our study is the modality of investigation by questionnaire, which cannot create the same stressful situation as might be experienced at the scene of the accident. Also it was requested that participants fill out the questionnaire without external help and within a time limit of 10 minutes. The study design does not allow assessment of the rate of compliance with these instructions.

Another limitation is the use of only one clinical scenario to evaluate our participants. Although better reliability might have been achieved with several cases, we aimed at obtaining a high response rate and therefore opted for a low time-consuming questionnaire. Menegazzi et al. found significant interrater agreement at higher GCS scores and only a moderate agreement at intermediate or low GCS values [25] whereas Rowley et al. found the best agreement in very high or very low GCS scores with greatest discrepancies in intermediate values [11]. We intentionally chose a clinical case of a severe traumatic head injury in the lower intermediate range, as studies have shown that there is a steep relationship between GCS 3 and 7 and mortality, followed by a shallower decline between 8 and 15 [26].

Finally, we do not know the level of training nor the distribution of specialties among the non responders to the questionnaire. This might possibly alter the conclusion.

## Conclusion

More than a third of the air-rescue physicians in Switzerland imprecisely scored the Glasgow coma scale in this study. Mistakes occur mainly in the assessment of the motor response followed by the verbal response while the eye component did not generate any wrong answers. In some cases, although the summed score was correct, it was calculated from incorrectly scored components. An association was found between performance and the level of training, with registrars producing more errors in scoring than their more experienced colleagues. However there was no difference between specialties.

This study indicates the need for education to reduce variability in GCS-Scoring. The GCS is an important score for clinical decision making and prognostication. Better training in prehospital scoring of the GCS is necessary.

## Competing interests

The authors declare that they have no competing interests.

## Authors' contributions

CH is the first author who designed the study, wrote the questionnaire, analyzed the results and wrote the manuscript. PS contributed substantially to the design of the study, it's coordination, it's analysis, the manuscript and the statistical approach. DRS contributed to the feasibility of the study and reviewed the manuscript and the statistical approach. NG contributed to the feasibility and coordination of the study and reviewed the manuscript text. All authors read and approved the final manuscript.

## Supplementary Material

Additional file 1 **Questionnaire**. The data provided represent the questionnaire sent to all participating helicopter bases. Click here for file
