# Supplementary material for: Knowledge of Glasgow coma scale by air-rescue physicians
Source: Scand J Trauma Resusc Emerg Med. 2009 Sep 1;17:39. doi: 10.1186/1757-7241-17-39 (PMC2743630; doi:10.1186/1757-7241-17-39)
Supplement: Additional file 1 — Questionnaire. The data provided represent the questionnaire sent to all participating helicopter bases. [file 1757-7241-17-39-S1.doc]

Helicopter base: ………………………………………..

Grade : Registrar [ ] Fellow [ ] Consultant [ ] Private practice [ ]

Specialty: Anesthesiology [ ] Internal medicine [ ] GP [ ] Others [ ]

Years of clinical experience: ……….......................................................................

Have you heard of the Glasgow Coma Scale?

Yes [ ] No [ ]

How many components contains the Glasgow Coma Scale?

1[ ] 2[ ] 3[ ] 4[ ] 5[ ]

List the components of the Glasgow Coma Scale …………………………………………………………………………………………………..

How many points belong to each single component? (Ex.: Component 1 = 9 points) …………………………………………………………………………………………………..

Minimal value obtainable in the Glasgow Coma Scale? …………………………………

Maximal value obtainable in the Glasgow Coma Scale?………………………………….

Clinical scenario:

37 year old patient who sustained a motor vehicle accident. Airways are clear, bilateral air entry. BP 110/85, HR 120/minute, Saturation 96%. No response to questioning, pinching of the right nipple elicits moaning and abnormal flexion of both arms. When you open his eyes, which are still closed, you notice pupils equal and reactive to light.

Glasgow Coma Scale: ............................

Points per component: ........./........./........../........../........../........../........
